# Data on compressibility of NBR samples with various cross-linking degree and zinc oxide content immersed in gasoline and oil

**DOI:** 10.1016/j.dib.2020.105470

**Published:** 2020-04-21

**Authors:** Ilya Karzov, Anton Nashchokin, Nikolay Tikhonov, Denis Kalugin, Artem Malakho

**Affiliations:** a Chemistry Department of Moscow State University, 1/11, Leninskie gory, Moscow, Russia, 119234; b Institute of New Carbon Materials and Technologies, 1/11, Leninskie gory, Moscow, Russia, 119234

**Keywords:** Nitrile butadiene rubber, Compressibility, Oil resistance, Ageing, Zinc oxide

## Abstract

NBR materials are widely used in oil refining, automotive industry and aviation due to the high tensile strength and elasticity, resistance to dilute acids. During operation with oils and fuels NBR materials undergo aging, which results in deterioration of the properties of materials [1]. The data on compressibility of NBR samples with 0, 1 and 10% zinc oxide and various cross-linking degree immersed in oil at 150°C for 5 hours and in gasoline at room temperature for 5 hours according to ASTM F146 – 12 [2] was collected. The cross-linking degree of NBR was calculated as the ratio of the heats of cure of samples vulcanized at specified temperate and time and unvulcanized samples as described at ISO 11357-5:2013 [3]. Compressibility was measured using a Tinius Olsen H5KS tensile testing machine according to ASTM F36 – 15 [4]. The data demonstrates the influence of vulcanized conditions and zinc oxide as a sulfur vulcanization activator content on compressibility change after deterioration in oil.

Specifications table

**Table:** 

Subject	Materials Chemistry
Specific subject area	The influence of cross-linking degree and zinc oxide content on the compressibility of NBR samples immersed in oil at 150°C and in gasoline at room temperature.
Type of data	Table
How data were acquired	Differential scanning calorimetry Compressibility measurement Instruments: NETZSCH DSC 204 Phoenix differential scanning calorimeter, NETZSCH Thermokinetics 3.0 software; Tinius Olsen H5KS tensile testing machine.
Data format	Raw Analyzed
Parameters for data collection	The weight and compressibility measurements were performed at room temperatures. The samples were immersed in oil at 150°C and in gasoline at room temperature for 5 hours. Differential scanning calorimetry runs were conducted under 100 ml/min nitrogen gas flow and the heating rates were 5 K/min. The size of test samples was 25 × 50 mm.
Description of data collection	Differential scanning calorimetry was carried out for prepared NBR samples and unvulcanised NBR samples. The cross-linking (conversion) extent was calculated as the ratio of the heats of cure of samples vulcanized at specified temperate and time and unvulcanized samples according to ISO 11357-5:2013. The assessment of oil resistance was carried out according to the ASTM F146 – 12 with immersion in oil 150°C and at gasoline at room temperature for 5 hours. The relative mass change and compressibility were measured according to ASTM F36 − 15.
Data source location	Chemistry Department of Moscow State University: Moscow Russia 55.702923, 37.523414
Data accessibility	Repository name: Mendeley data Direct URL to data: https://data.mendeley.com/datasets/2hskypf8ch/1 DOI: 10.17632/2hskypf8ch.1 http://dx.doi.org/10.17632/2hskypf8ch.2#folder-8a543e34-4102-41ae-a5e1-d6d0dc0da992 http://dx.doi.org/10.17632/2hskypf8ch.2#folder-155da583-05ce-408a-b927-017969ddd5e6

## Value of the data

• The data on compressibility demonstrates the influence of vulcanized conditions and zinc oxide as a sulfur vulcanization activator content on NBR resistance to aging in oil. It may be used for the optimization of zinc oxide content and curing time in NBR preparation synthesis.
• The data in the article may be used in further experiments to identify the key factors of NBR preparation responsible for oil resistance.
• The data may be used for research of NBR aging mechanism in fuels which includes a set of parallel processes [5].

## 1 Data Description

The composition of all blends are shown in Table 1. K1, K2 and K3 blends have different zinc oxide content.

**Table 1:** Compositions of NBR-blends.

Blends	Composition, phr*				Name of hosted file in repository, URL
	S	ZnO	TMTD	MBT	
K1	2	0	4	1	Table 1. Compositions of NBR-blends http://dx.doi.org/10.17632/2hskypf8ch.2#file-610b580d-bfaa-4118-bd1b-448a5d47a2dd
K2	2	1	4	1	
K3	2	10	4	1	

⁎ phr – parts per hundred of rubber PBNK-40

Vulcanization conditions (temperature and time) are shown in Table 2.

**Table 2:** Vulcanization conditions (temperature, time) for NBR samples.

Sample	Vulcanization time, min	Vulcanization temperature, °С	Name of hosted file in repository, URL
K1-1	26	170	Table 2. Vulcanization conditions (temperature, time) for NBR samples http://dx.doi.org/10.17632/2hskypf8ch.2#file-b74512f2-57aa-4e70-885d-a4dbd486ce64
K1-2	47	170	
K1-3	120	170	
K2-1	10	140	
K2-2	16	140	
K2-3	75	140	
K3-1	7	140	
K3-2	12	140	
K3-3	50	140	

Table 3 represents the calculation results on cross-linking extent for K1, K2 and K3 blends vulcanized according Table 2, on compressibility before and after immersion in gasoline and oil and mass change. All raw data are presented in Tables 4, 5 and 6 (data on compressibility, mass and DSC respectively).

**Table 3:** NBR compressibility before and after aging in oil and gasoline, the relative change in mass and cross-linking extent, calculated by as the ratio of the heats of cure of samples vulcanized (vulcanisation conditions are presented at table 2) and unvulcanized samples.

Samples	Cross-linking extent, %	Compressibility, %	Gasoline		Oil, 150°С		Name of hosted file in repository, URL
			Compressibility, %	Δm/m, %	Compressibility, %	Δm/m, %	
K1-1	70	54±4	51±4	−0,4	74±2	−15	Table 3. NBR compressibility before and after aging http://dx.doi.org/10.17632/2hskypf8ch.2#file-15b3b95b-66f0-4a80-bdf8-ad1b74c00ab5
K1-2	95	54±3	48±3	−0,3	76±1	−15	
K1-3	99	53±2	48±2	−0,2	74±3	−12	
K2-1	47	32±1	31±1	−0,4	62±4	−10	
K2-2	85	30±1	31±1	−0,3	56±2	−11	
K2-3	96	34±2	30±2	−0,3	68±4	−11	
K3-1	75	54±1	37±1	−0,2	55±1	−7	
K3-2	81	41±2	26±2	−0,1	56±2	−8	
K3-3	97	28±3	29±3	−0,2	57±1	−8

**Table 4:** Raw data of NBR compressibility.

Samples	Compressibility before immersion, %	Compressibility after immersion in gasoline, %	Compressibility after immersion in oil at 150°С, %	Name of hosted file in repository, URL
K1-1	51,2 55,8 49,2 59,9 53,7	46,2 53,0 54,1	72,1 73,4 75,3	Table 4. Raw data of NBR compressibility http://dx.doi.org/10.17632/2hskypf8ch.2#file-2f52310c-e9a5-4bf9-9c87-107c9b39e1ac
K1-2	50,4 55,3 58,1 52,6 54,4	46,5 51,6 46,2	76,7 74,3 75,4	
K1-3	54,2 52,8 50,9	49,9 46,1 46,9	76,8 70,3 73,6 74,7	
K2-1	32,4 31,7 31,2	31,0 32,4 29,9	61,6 65,9 58,8	
K2-2	29,2 29,3 31,0	29,6 32,1 31,0	57,1 55,0 55,2	
K2-3	34,8 34,2 32,2	30,5 32,0 27,8	72,3 68,8 64,3	
K3-1	53,6 52,8 54,9	37,8 36,2 36,6	55,0 55,7 54,7	
K3-2	43,4 38,9 39,9	27,9 24,6 26,4	53,5 57,3 56,1	
K3-3	31,6 25,9 26,8	32,1 28,1 26,7	57,8 56,8 56,4

**Table 5:** Raw data of NBR relative change in mass before and after immersion in gasoline and oil.

Samples	Mass before immersion in gasoline, %	Mass after immersion in gasoline, %	Compressibility before immersion in oil at 150°С, %	Compressibility after immersion in oil at 150°С, %	Name of hosted file in repository, URL
K1-1	3,881	3,874	4,114	3,816	Table 5. Raw data of NBR relative change in mass before and after immersion in gasoline and oil http://dx.doi.org/10.17632/2hskypf8ch.2#file-a4649a2c-6b8b-4c77-9179-10ab6dffeb50
K1-2	4,196	4,190	4,396	4,040	
K1-3	4,922	4,914	4,550	4,182	
K2-1	3,471	3,457	3,576	3,222	
K2-2	3,614	3,602	5,030	4,454	
K2-3	3,680	3,670	3,878	3,460	
K3-1	5,988	5,970	6,160	5,237	
K3-2	6,332	6,314	6,657	5,685	
K3-3	6,879	6,863	7,325	6,426	

## 2 Experimental Design, Materials, and Methods

The mixing of rubber compounds was carried out in two steps. In the first step, sulfur, TMTD, MBT and zinc oxide were mixed with toluene under constant stirring at room temperature for 30 min. In the second step, the powder acrylonitrile-butadiene rubber was added and the blend was stirred for 12 h. The ratio PBNK-40 : toluene was 1:4. The composition of all blends are shown in Table 1. Afterwards the blend was placed on the silicone paper to form an even layer and dried out for 48h.

The vulcanized sheets (from which test specimens were cut) were produced in an electrically heated hydraulic press. The pressure was 4 MPa, vulcanization temperature and time are shown in Table 2.

Differential scanning calorimetry was carried out on 10 mg samples of each material using a NETZSCH DSC 204 Phoenix differential scanning calorimeter calibrated with metal standards. Runs were conducted under 100 ml/min nitrogen gas flow and the heating rate was 5 K/min. Kinetic parameters of the resins curing processes were calculated on the base of differential scanning calorimetry data via NETZSCH Thermokinetics 3.0 software. The cross-linking (conversion) extent was calculated as the ratio of the heats of cure of samples vulcanized at specified temperate and time and unvulcanized samples according to ISO 11357-5:2013.

The assessment of oil resistance was carried out according to the following procedure. Test samples 25 × 50 mm in size were weighed and immersed in a flask with oil at a temperature of 150°C for 5 hours. Then it was kept for 30 minutes in oil at room temperature, washed in nephras and the weight was re-measured. The aging in gasoline was carried out at room temperature for 5 hours. The immersion in oil and gasoline was carried out according to according to ASTM F146 – 12. Compressibility was measured using a Tinius Olsen H5KS tensile testing machine according to ASTM F36 − 15.

## CRediT authorship contribution statement

**Ilya Karzov:** Data curation, Investigation, Writing - original draft. **Anton Nashchokin:** Writing - review & editing. **Nikolay Tikhonov:** Methodology, Software. **Denis Kalugin:** Software, Validation. **Artem Malakho:** Conceptualization, Supervision.

## Declaration of Competing Interest

The research was supported by the Ministry of Education and Science of the Russian Federation, Resolution No. 218, 2010, April 9-th (Contract No. 03.G25.31.0220 (Development of high-temperature composite seals for improve energy-saving and reliability of sealing equipment and pipelines) between JSC UNICHIMTEK and Lomonosov Moscow State University).

The authors declare that they have no known competing financial interests or personal relationships which have, or could be perceived to have, influenced the work reported in this article.

## Appendix Supplementary materials

Image, application 1
